# Psychometric properties of the Khmer version of the locomotor capabilities index (LCI-5) for lower-limb prosthesis and orthosis users

**DOI:** 10.1097/PXR.0000000000000504

**Published:** 2025-10-23

**Authors:** Nerrolyn Ramstrand, Alan Maddock, Thearith Heang, Nil Ean, Sisary Kheng

**Affiliations:** 1 Department of Rehabilitation, School of Health and Welfare, Jönköping University, Jönköping, Sweden; 2 Department of Health Psychology, School of Population Health, Royal College of Surgeons in Ireland, Dublin, Ireland; 3 Exceed Worldwide, Phnom Penh, Cambodia; 4 Department of Prosthetics and Orthotics, National Institute of Social Affairs, Phnom Penh, Cambodia; 5 The Center for Trauma Care and Research Organization (CTRO), Phnom Penh, Cambodia; 6 Department of Psychology, Royal University of Phnom Penh, Phnom Penh, Cambodia

**Keywords:** psychometrics, validation, mobility, LCI-5, outcome measure, prosthetic limb, orthotic devices, LMIC

## Abstract

**Background:**

The unavailability of validated outcome measures in local languages limits the extent to which the impact of prosthetic and orthotic devices can be assessed.

**Objectives:**

This study examined the psychometric properties of the Khmer locomotor capabilities index (LCI-5) for use with lower-limb prosthesis users. An additional aim was to explore its psychometric properties when used on lower-limb orthosis users.

**Study Design:**

A cross-sectional design was used.

**Methods:**

The Khmer version of the LCI-5 was administered to clients attending 3 prosthetic and orthotic clinics in urban and rural Cambodia. Internal consistency and floor and ceiling effects were evaluated. Exploratory Factor Analysis and Confirmatory Factor Analysis (CFA) were performed on independent samples of prosthesis user data. A separate CFA was conducted on a data from orthosis users.

**Results:**

Four hundred thirty-seven clients completed the LCI-5. The data had high internal consistency; however, a significant ceiling effect was observed. For prosthesis users, the optimal solution arising from exploratory Factor Analysis and CFA was a 2-factor model. A modified 2-factor model produced an acceptable fit for orthosis users.

**Conclusions:**

The results present a 2-factor structure of the Khmer LCI-5, which can be used to assess mobility in prosthesis and orthosis users. It is important to note that the factor structure of the Khmer version is not consistent with the English version. Additional work is required to address ceiling effects associated with the Khmer LCI-5.

## Introduction

The Kingdom of Cambodia has a long history of civil unrest, peaking during the Khmer Rouge era from 1975 to 1979, but remaining unstable until 1993.^1^ As a result of mass violence inflicted by the Khmer Rouge, it is estimated that around one-quarter of the population perished, while many who survived were left with permanent disability. Accordingly, Cambodia has experienced some of the highest rates of disability in the world.^2^ It has been estimated that 9.6% of the Cambodian population aged 5 years or older has a disability affecting their mobility.^3^ Although weapon trauma and conflict have historically accounted for a major proportion of these disabilities, there has now been a shift toward disabilities caused by noncommunicable disease and traffic accidents.^4^ In Cambodia, women make up 57% of the general population with disabilities,^3^ yet 83% of people accessing prosthetic and orthotic services are men.^4^ Eighty-four percent of people with disabilities live in rural areas with limited access to rehabilitation services.^5^

Assistive technologies, such as prostheses and orthoses, are often used to reduce impairment caused by mobility disabilities. In the Cambodian context, access to prosthetic or orthotic devices has been qualitatively reported in to have positive effects on mobility of people with physical impairments.^6^ To date, there is no quantitative research to support these findings, likely due to the unavailability of validated measures of mobility in Khmer, the native language of Cambodia. The lack of quantitative data is typical of the situation in many low- and middle-income countries, where the availability of validated patient-reported outcome measures (PROMs) are limited.^7^ Malapati et al^7^ highlight this and identify specific barriers to the use of PROMs in low- and middle-income countries. Issues lifted by this group, including limited availability of translation versions, culturally irrelevant items and low-literacy levels, are all relevant in the Cambodian context.

The Locomotor Capabilities Index (LCI) is a self-report measure of mobility that was initially developed to assess the ability of lower-limb prosthesis users to perform activities with their prosthetic device (e.g., get up from a chair; walk while carrying an object).^8^ The instrument was initially developed as part of the Prosthetic Profile of the Amputee questionnaire^8^ but has since been widely accepted as a stand-alone measure.^9,10^ Literature reviews have reported the LCI-5 to be one of the most commonly applied PROMs for major lower-limb amputation,^10,11^ with reports of the measure being use on transfemoral and transtibial prosthesis users as well as on people who have been amputated for dysvascular and traumatic reasons.^11^ The original version of the LCI listed 14 activities to be rated on a 4-point ordinal scale ranging from 0, not able to do the activity to 3, able to accomplish the activity alone. The index was later modified to include 5 response items, with the original option, “able to accomplish the activity alone” (3 points) divided into 2 distinct response options: “alone with ambulation aids” and “alone without ambulation aids.”^12^ In psychometric testing, the 5-level version (LCI-5) was found to have a lower ceiling effect and larger effect size than the original 4-level index.^12^ A composite measure of overall locomotor ability is obtained from the LCI-5 by summing the individual scores obtained for each of the 14 items. The index can also be divided into two 7-item subscales which represent basic (items 1, 4, 5, 8-11) and advanced activities (items 2, 3, 6, 7, 12‒14).^12,13^

The English version of the LCI-5 has been reported as having good internal consistency, test-rest reliability, construct validity, convergent validity, sensitivity to change, and responsiveness.^12,14,15^ To date, the LCI-5 has been translated and validated into Swedish,^16^ Finnish,^17^ Persian,^18^ and German.^12^ In the Finnish version, a one-factor model was found to explain 83% of the total variance.^17^ Caution was advised in using the Persian version of the LCI-5 with physically active and young patients due to high ceiling effects.

The LCI-5 is considered an interesting self-report measure of mobility in the Cambodian context as it covers a range of items which have been identified by Cambodian prosthesis and orthosis users as being challenging to perform, that is, walking, carrying objects, and getting up from the floor after sitting or kneeling.^6^ A rigorous translation and cultural validation of the LCI-5 into Khmer have been published,^19^ but psychometric testing of the Khmer version has not yet been reported. Although the original version of the LCI-5 was developed for prosthesis users, the Khmer version was adapted so that it could be used for people wearing lower-limb orthotic devices as well.^19^ For example, the instruction “would you say that you are able to do the following activities with your prosthesis on” was adapted to read “would you say you are able to do the following activities while wearing your assistive device.” The authors of this adapted version highlight that “care should be taken in future studies to evaluate psychometric properties when it is applied on lower-limb orthosis users.”^19^

The quality of any PROM is dependent on its psychometric properties, which provide evidence that they measure what they are intended to measure (validity) and can reproduce results consistently in time and space (reliability).^20^ Items included within a PROM should assess a single construct (internal consistency) and should detect changes in a person's condition (responsiveness) without too many people responding at extremely high or low ends of the spectrum (ceiling/floor effects).^20^ As psychometric properties are closely linked to the population that they are intended for, it is important to investigate psychometric properties when PROMs are used on the specific population of interest.^21^ Given that the Khmer version of the LCI-5 has been adapted for use on people with either prosthetic or orthotic device,^19^ there is a need to investigate psychometric properties of the instrument for both subgroups.

### Aim

This study aimed to examine the psychometric properties (floor/ceiling effects, internal consistency, and structural validity) of the Khmer version of the LCI-5 with lower-limb prosthesis users and to explore its potential as a measure of mobility in lower-limb orthosis users.

## Methods

### Research design

This study used a cross-sectional design. Data required to examine the validity of the LCI-5 was collected using a questionnaire administered to clients attending any 1 of 3 prosthetic and orthotic clinics in Cambodia (1 urban and 2 rural clinics). The questionnaire was part of a larger study investigating determinants of mental health in Cambodian prosthesis and orthosis users.^22^ Data collection took place between March 2023 and July 2024.

### Participants

Questionnaire respondents were included in this study if they were older than 18 years and used a lower limb prosthetic or orthotic device. Those with prosthetic devices were included if they used a trans-tibial prosthesis, knee disarticulation prosthesis, trans-femoral prosthesis, or hip disarticulation prosthesis. Respondents with orthotic devices were included if they used an ankle-foot-orthosis, knee-ankle-foot-orthosis, or hip-knee-ankle-foot-orthosis. People using multiple orthotic devices or multiple prosthetic devices were included. Respondents were excluded from the study if they used a prosthetic device on one leg and an orthotic device on their contralateral leg as it would not have allow the researchers to distinguish data between device types.

### Data collection

Data were collected between March 2023 and July 2024, with participants completing the questionnaire using a digital form generated in Qualtrics. In addition to the Khmer version of the LCI-5,^19^ the questionnaire included items related to the clients' demographics (sex, age, and participant location), social situation (education, employment, and income), physical status (type of disability, cause of disability, and pain), and the type of assistive device that they used (prosthesis/orthosis). If duplicate data were identified from the same client, the first data collection was retained, while any additional data from the same client were removed. Ethical approval for the study was obtained from the National Ethics Committee for Health Research in Cambodia (#207 NEHCR).

### Data analysis

Descriptive data were analyzed using IBM SPSS Statistics (v28). Potential floor and ceiling effects were evaluated by calculating skewness and kurtosis of the data as well as the percentage of responses falling into the lowest (0) and highest (5) response ranges. Cronbach alpha was calculated as a measure of internal consistency. Structural equation modelling was performed using RStudio, Build 4.1.2.^23^ As the LCI-5 was developed to evaluate levels of mobility in prosthesis users, Exploratory Factor Analysis (EFA) and Confirmatory Factor Analysis (CFA) were initially performed on a sample which included only prosthesis users. To protect against overfitting, EFA and CFA were performed on different data sets by randomly dividing the sample into two.^24^ Exploratory Factor Analysis was completed on the first prosthesis user data set (sample n = 173), while separate CFAs were then conducted on the second prosthesis user data set (n = 172). Separate CFAs were then conducted on the data set which included only orthosis users (n = 92).

### EFA

To test that the assumptions for conducting an EFA, a correlation matrix was generated to assess for linear dependencies. The Bartlett test of sphericity was used to assess for factorability, and the Kaiser-Meyer-Olkin measure was applied to measure sampling adequacy. The EFA was conducted in RStudio using EFA.dimensions 0.1.8.4^25^ and the Laavan 0.6-18^26^ packages. We used a GEOMIN rotation, assuming that the factors would be correlated and, as such, an oblique rotation method would be most appropriate.^27^ Data were extracted using principal axis factor extraction. One incomplete measure was removed from the analysis.

To determine the number of factors to retain in the model, we used 3 different approaches: (1) eigenvalue method, retaining any factors with an eigenvalue above 1^28^; (2) visual scree plot, retaining factors above the distinct point of break in the slope of the plot^29^; and (3) parallel analysis, retaining factors where the eigenvalues extracted from real data exceed those extracted from random data.^29^ To establish the adequacy of the factor model, a factor loading cutoff value equal or exceeding 0.37 was established.^30^ In accordance with recommendation made by Hair et al, in 2010,^31^ a minimum sample size was calculated using a ratio of 10 participants per question (i.e., 10 × 14 =140).

### CFA

Confirmatory Factor Analysis was conducted using structural equation modelling with estimates of model parameters generated using maximum likelihood estimation. Three alternative models were specified and estimated.

Model 1: A single general factor model.

Model 2: A 2-factor model, specifying the existing 2 LCI-5 subscales representing basic (items 1, 4, 5, 8‒11) and advanced activities (items 2, 3, 6, 7, 12‒14).^12^

Model 3: A model specifying the best solution achieved in EFA.

Modification indexes were used to identify components in the model that could be fine-tuned for the purpose of improving overall goodness-of-fit (post hoc model modification).^32^

### Assessment of model Fit

In both the EFA and CFA analyses, comparative fit of models were assessed using Akaike information criteria (AIC)^33^ and Bayesian information criteria (BIC).^34^ The BIC is recognized as a good indicator of comparative model fit.^35,36^ The model having the lowest value of AIC and BIC was considered to have the best fit. Goodness of fit was assessed in line with guidelines set out by Hoyle and Panter^37^ with a range of fit indices used, including: chi-square (χ^2^), Comparative Fit Index (CFI),^38^ and Tucker-Lewis index (TLI).^39^ A nonsignificant chi-square and values greater than 0.90 for the TLI and CFI are considered to reflect acceptable model fit. The Root Mean Square Error of Approximation (RMSEA)^40^ was also assessed, with values below 0.05 indicating close fit and values up to 0.08 indicating reasonable fit in the population.^41^ Finally, the Standardized Root Mean Square Residual (SRMR)^42^ was calculated as it has been recommended for its sensitivity to model misspecification.^43^ Standardized Root Mean Square Residual values less than 0.08 were considered to represent an acceptable model fit.^44^

## Results

Demographic data for the 437 participants who completed the Khmer version of the LCI-5 are presented in Table 1. Most respondents were male (80%) with a relatively low level of education (50% primary education or no education). Most were aged older than 35 years (n = 381; 82%). In total, 79% were lower-limb prosthesis users (n = 346), while the remaining 21% were lower-limb orthosis users (n = 91). Descriptive statistics associated with participant responses on the translated LCI-5 measure are presented in Table 2. Items 1‒6 recorded very high negative skewness, while items 1‒5 had extremely high kurtosis. A very high percentage of responses was at the highest end of the response scale, indicating a significant ceiling effect.^45^ Cronbach alpha was calculated at 0.89 reflecting a high level of internal consistency.^46^

**Table 1.**
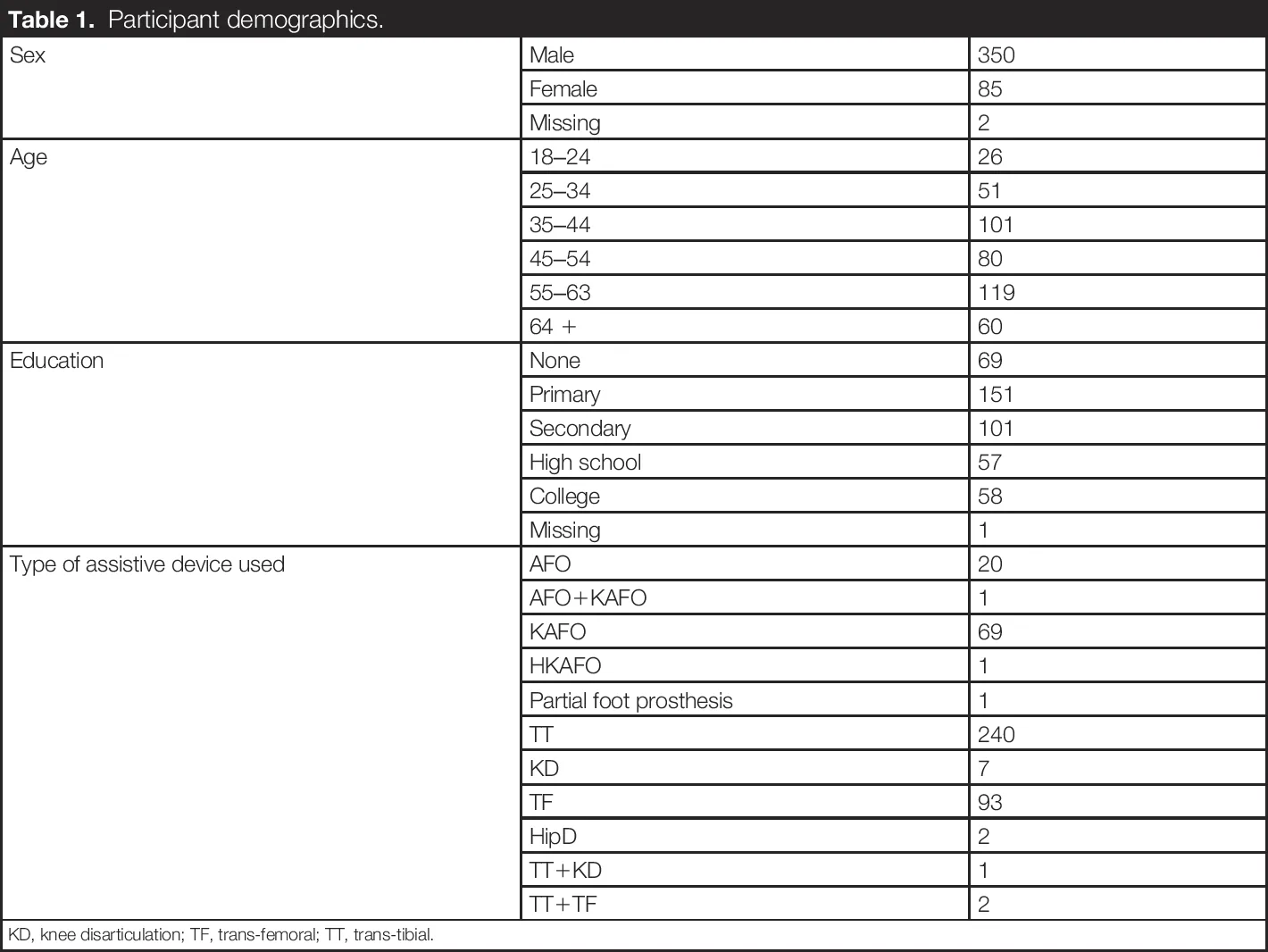
Participant demographics.

Sex	Male	350
	Female	85
	Missing	2
Age	18‒24	26
	25‒34	51
	35‒44	101
	45‒54	80
	55‒63	119
	64 +	60
Education	None	69
	Primary	151
	Secondary	101
	High school	57
	College	58
	Missing	1
Type of assistive device used	AFO	20
	AFO+KAFO	1
	KAFO	69
	HKAFO	1
	Partial foot prosthesis	1
	TT	240
	KD	7
	TF	93
	HipD	2
	TT+KD	1
	TT+TF	2

KD, knee disarticulation; TF, trans-femoral; TT, trans-tibial.

**Table 2.**
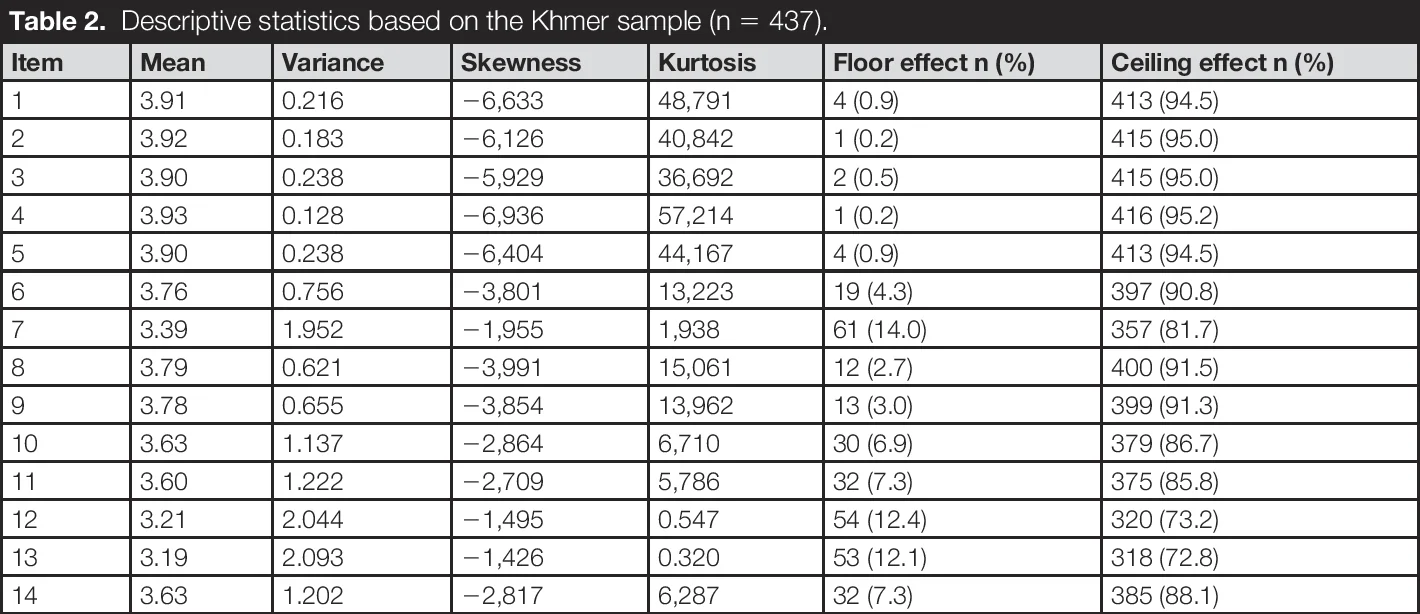
Descriptive statistics based on the Khmer sample (n = 437).

Item	Mean	Variance	Skewness	Kurtosis	Floor effect n (%)	Ceiling effect n (%)
1	3.91	0.216	−6,633	48,791	4 (0.9)	413 (94.5)
2	3.92	0.183	−6,126	40,842	1 (0.2)	415 (95.0)
3	3.90	0.238	−5,929	36,692	2 (0.5)	415 (95.0)
4	3.93	0.128	−6,936	57,214	1 (0.2)	416 (95.2)
5	3.90	0.238	−6,404	44,167	4 (0.9)	413 (94.5)
6	3.76	0.756	−3,801	13,223	19 (4.3)	397 (90.8)
7	3.39	1.952	−1,955	1,938	61 (14.0)	357 (81.7)
8	3.79	0.621	−3,991	15,061	12 (2.7)	400 (91.5)
9	3.78	0.655	−3,854	13,962	13 (3.0)	399 (91.3)
10	3.63	1.137	−2,864	6,710	30 (6.9)	379 (86.7)
11	3.60	1.222	−2,709	5,786	32 (7.3)	375 (85.8)
12	3.21	2.044	−1,495	0.547	54 (12.4)	320 (73.2)
13	3.19	2.093	−1,426	0.320	53 (12.1)	318 (72.8)
14	3.63	1.202	−2,817	6,287	32 (7.3)	385 (88.1)

### Exploratory factor analysis

The Bartlett test of sphericity indicated that the correlation matrix was not random χ^2^ (91) = 1888.71, *p* ≤ 0.001. The Kaiser-Meyer-Olkin measure of sampling adequacy was 0.81, considered as meritorious for conducting EFA.^47^ Existing research conducted on the English version of the LCI-5^12^ indicates that a single-factor solution or a two-factor solution may be considered optimal. Parallel analysis of our data suggested that 2 factors should be retained (Supplemental Digital Content 1, supplementary table 1, http://links.lww.com/POI/A350), while the eigenvalue method (Supplemental Digital Content 1, supplementary data 2, http://links.lww.com/POI/A350) and the scree plot (Supplemental Digital Content 1, supplementary data 3, http://links.lww.com/POI/A350) indicated that a 3-factor model may be more appropriate. Consequently, solutions for 1–4 factor models were explored. Poor model fit was evident across all initial solutions (Table 3). Further iterations were conducted to identify an optimal solution, with factor loadings, communalities, and fit indices examined for each model.

**Table 3.**
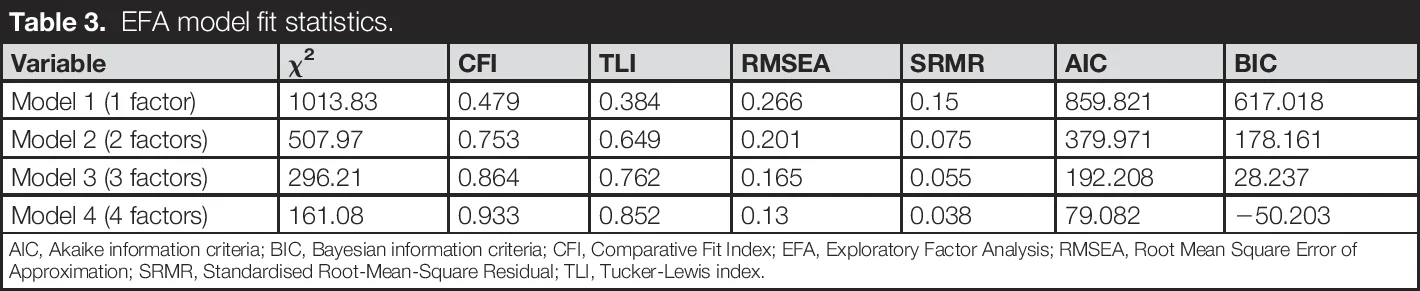
EFA model fit statistics.

Variable	χ^2^	CFI	TLI	RMSEA	SRMR	AIC	BIC
Model 1 (1 factor)	1013.83	0.479	0.384	0.266	0.15	859.821	617.018
Model 2 (2 factors)	507.97	0.753	0.649	0.201	0.075	379.971	178.161
Model 3 (3 factors)	296.21	0.864	0.762	0.165	0.055	192.208	28.237
Model 4 (4 factors)	161.08	0.933	0.852	0.13	0.038	79.082	−50.203

AIC, Akaike information criteria; BIC, Bayesian information criteria; CFI, Comparative Fit Index; EFA, Exploratory Factor Analysis; RMSEA, Root Mean Square Error of Approximation; SRMR, Standardised Root-Mean-Square Residual; TLI, Tucker-Lewis index.

The optimal solution arising from the EFA process was a modified 2-factor model. During the process of exploring additional iterations, item 8, go upstairs with a handrail, and item 9, go downstairs with a handrail were identified as having a large degree of covariance. So too did items 10, step up a curb; item 11, step down a curb; item 12, go up a few stairs without a handrail; and item 13, go down a few stairs without a handrail. After testing various iterations, which included allowing covariances between these pairs of items, the decision was ultimately made to remove items 8, 10, and 12. Item 6, walk outside on uneven ground, was also removed due to high error variance and a low standardized loading. Item 5, walk outside on even ground, was found to have significant loadings on both factors. As item 5 was considered to be conceptually related to both factors 1 and 2, the decision was made to allow for cross-loading of this item.

The final 2-factor, 10 item, model was considered to fit the data very well χ^2^ = 44.50; df = 33; *p* = 0.087; CFI = 0.988; TLI = 0.984; RMSEA = 0.045; SRMR = 0.055). The chi-square value was nonsignificant, and the CFI, TLI, RMSEA, and SRMR all met the criteria for acceptable fit. Factor loadings for this modified 2-factor model, highlighting which items reflect which factor, are presented in Table 4.

**Table 4.**
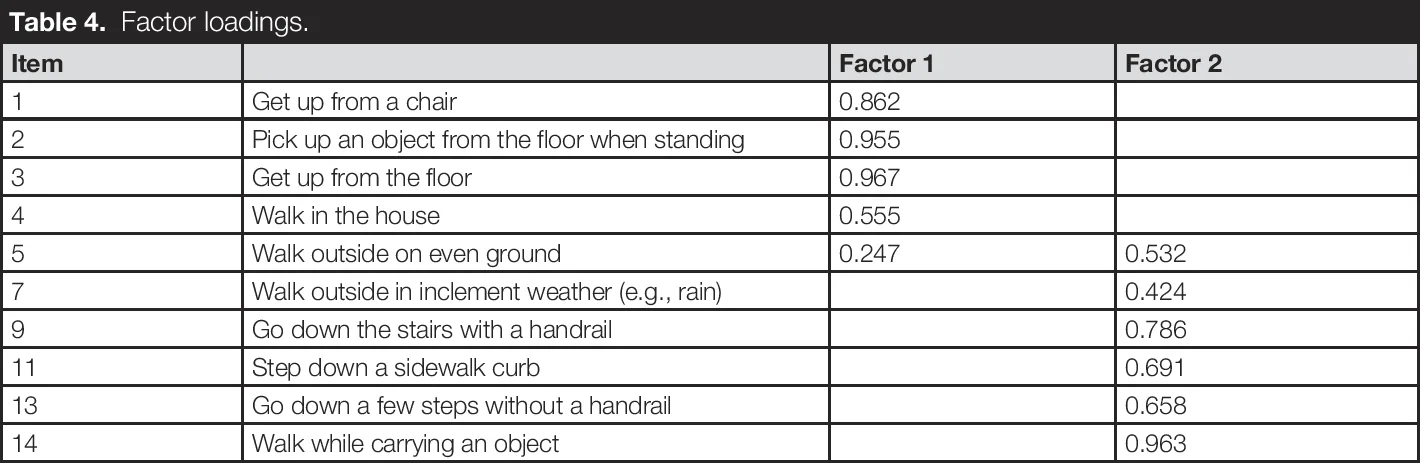
Factor loadings.

Item		Factor 1	Factor 2
1	Get up from a chair	0.862	
2	Pick up an object from the floor when standing	0.955	
3	Get up from the floor	0.967	
4	Walk in the house	0.555	
5	Walk outside on even ground	0.247	0.532
7	Walk outside in inclement weather (e.g., rain)		0.424
9	Go down the stairs with a handrail		0.786
11	Step down a sidewalk curb		0.691
13	Go down a few steps without a handrail		0.658
14	Walk while carrying an object		0.963

### Confirmatory factor analysis

Confirmatory Factor Analysis was undertaken to specify and test 3 alternative models on previously unused data sets (i.e., the second data set drawn from prosthesis users and the data set including only users of orthotic devices). The 3 alternative models tested were (1) a single factor solution (model 1), (2) a 2-factor model based on the original subscales of the English version of LCI-5^12^ (model 2), and (3) the modified 2-factor solution identified using EFA (model 3).

### CFA—prosthesis users

During initial exploration of the data, it was observed that items 2 and 4 were perfectly correlated. As a result, item 2, picking up an object from the floor, was removed from all CFA models. The results for each of the 3 models tested are presented in Table 5. Model 3 (modified 2-factor model) was adapted to allow for covariance between items 9 and 11 as the modification index indicated a high degree of covariance between these items (30.84). Cross-loading of item 5 was on factor 2 was removed.

**Table 5.**
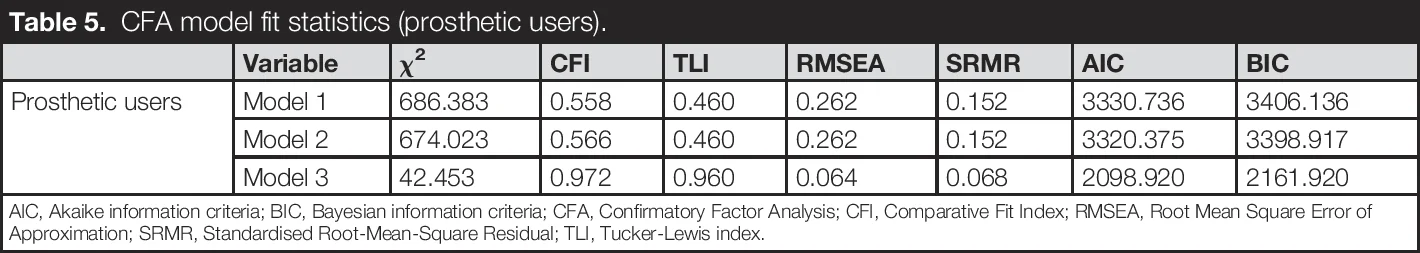
CFA model fit statistics (prosthetic users).

	Variable	χ^2^	CFI	TLI	RMSEA	SRMR	AIC	BIC
Prosthetic users	Model 1	686.383	0.558	0.460	0.262	0.152	3330.736	3406.136
	Model 2	674.023	0.566	0.460	0.262	0.152	3320.375	3398.917
	Model 3	42.453	0.972	0.960	0.064	0.068	2098.920	2161.920

AIC, Akaike information criteria; BIC, Bayesian information criteria; CFA, Confirmatory Factor Analysis; CFI, Comparative Fit Index; RMSEA, Root Mean Square Error of Approximation; SRMR, Standardised Root-Mean-Square Residual; TLI, Tucker-Lewis index.

The CFA process found that the modified 2-factor model (model 3) had the lowest AIC and BIC and was the best fit for the data (χ^2^ = 42.45; df = 25; *p* = 0.016; CFI = 0.972; TLI = 0.960; RMSEA = 0.064; SRMR = 0.068). Although the chi-square value in this model was significant, the CFI, TLI, RMSEA, and SRMR all met the criteria for acceptable fit.^37^ Factor loadings for this model are presented in Table 6. The Khmer version of the LCI-5 reflecting this model is presented as supplementary file 4, Supplemental Digital Content 1, http://links.lww.com/POI/A350.

**Table 6.**
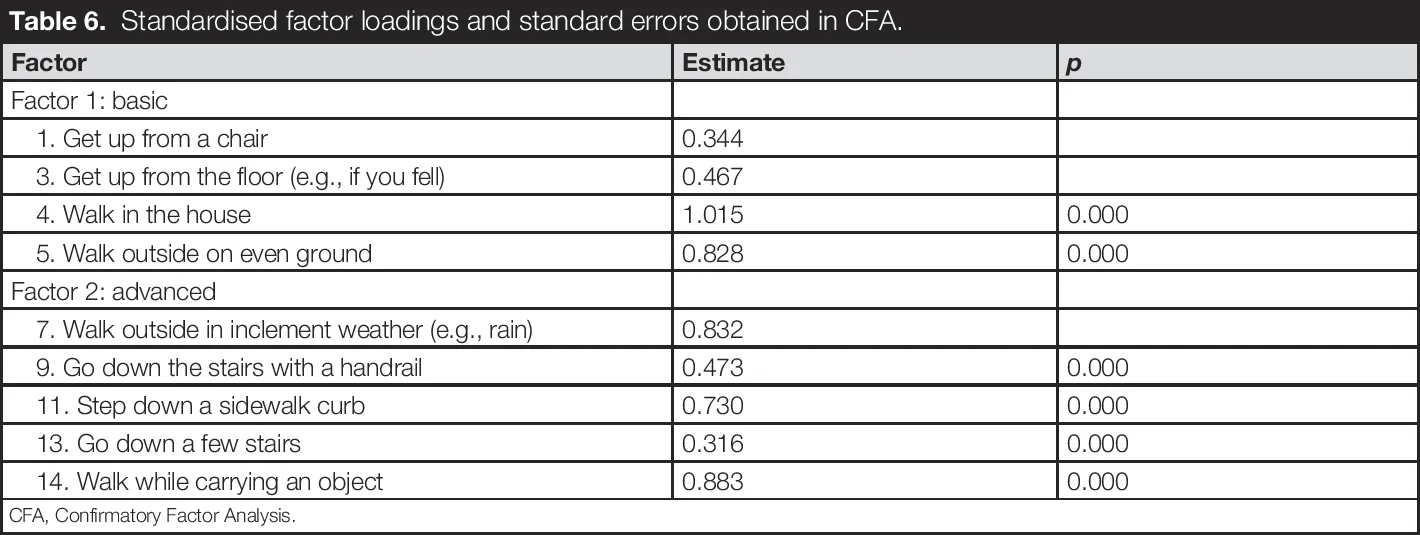
Standardised factor loadings and standard errors obtained in CFA.

Factor	Estimate	*p*
Factor 1: basic		
1. Get up from a chair	0.344	
3. Get up from the floor (e.g., if you fell)	0.467	
4. Walk in the house	1.015	0.000
5. Walk outside on even ground	0.828	0.000
Factor 2: advanced		
7. Walk outside in inclement weather (e.g., rain)	0.832	
9. Go down the stairs with a handrail	0.473	0.000
11. Step down a sidewalk curb	0.730	0.000
13. Go down a few stairs	0.316	0.000
14. Walk while carrying an object	0.883	0.000

CFA, Confirmatory Factor Analysis.

### Orthosis users

During exploration of the data set which included only lower-limb orthosis users, it was observed that items 10 and 11 were perfectly correlated. As a result, item 10 was removed from all models. The results are presented in Table 7. Model 3 (modified 2-factor model) was adapted to allow for covariance between items 1 and 4 and 7 and 11 as the modification index indicated a high degree of covariance between these items (18.77: 15.62, respectively). Cross-loading of item 5 on factor 2 was removed due to low factor loading (−0.017), while cross-loading was added for item 9 as the modification index indicated that it had a strong relationship with factor 1 (17.5).

**Table 7.**
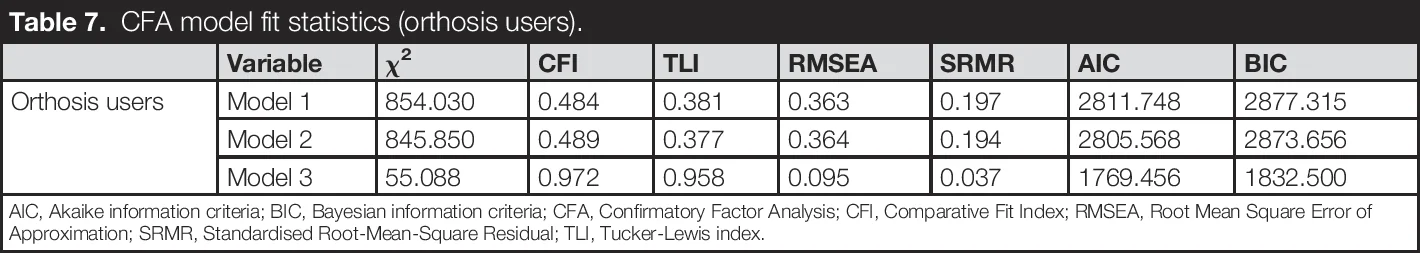
CFA model fit statistics (orthosis users).

	Variable	χ^2^	CFI	TLI	RMSEA	SRMR	AIC	BIC
Orthosis users	Model 1	854.030	0.484	0.381	0.363	0.197	2811.748	2877.315
	Model 2	845.850	0.489	0.377	0.364	0.194	2805.568	2873.656
	Model 3	55.088	0.972	0.958	0.095	0.037	1769.456	1832.500

AIC, Akaike information criteria; BIC, Bayesian information criteria; CFA, Confirmatory Factor Analysis; CFI, Comparative Fit Index; RMSEA, Root Mean Square Error of Approximation; SRMR, Standardised Root-Mean-Square Residual; TLI, Tucker-Lewis index.

The CFA process found model 3 to be an acceptable fit; it also reflected the lowest AIC and BIC, again indicating that from the models tested, this is the model of best fit for the data. The results of this CFA are presented in Table 8. The Khmer version of the LCI-5 reflecting this model is presented as supplementary file 5, Supplemental Digital Content 1, http://links.lww.com/POI/A350.

**Table 8.**
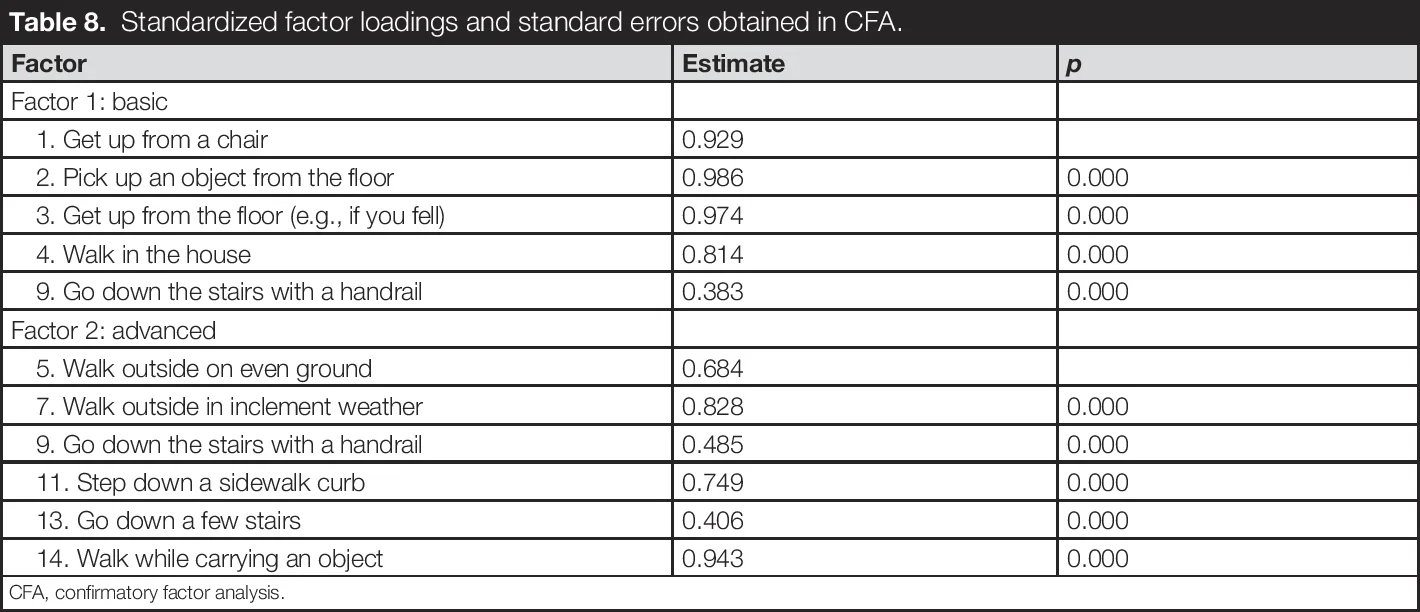
Standardized factor loadings and standard errors obtained in CFA.

Factor	Estimate	*p*
Factor 1: basic		
1. Get up from a chair	0.929	
2. Pick up an object from the floor	0.986	0.000
3. Get up from the floor (e.g., if you fell)	0.974	0.000
4. Walk in the house	0.814	0.000
9. Go down the stairs with a handrail	0.383	0.000
Factor 2: advanced		
5. Walk outside on even ground	0.684	
7. Walk outside in inclement weather	0.828	0.000
9. Go down the stairs with a handrail	0.485	0.000
11. Step down a sidewalk curb	0.749	0.000
13. Go down a few stairs	0.406	0.000
14. Walk while carrying an object	0.943	0.000

CFA, confirmatory factor analysis.

## Discussion

A global shift toward evidence-based practice and value-driven health care has meant that PROMs are increasingly being required as a central component of good clinical care.^48-50^ Patient-reported outcome measures provide health care service providers with standardized objective data to support clinical decisions, assess the effectiveness of interventions, and help to support allocation of resources. Although many measures are available to assess the health status of clients receiving prosthetic and orthotic services, there remain major barriers to their global uptake, including a lack of availability in local languages, insufficient cross-cultural adaptation, and unknown psychometric properties of translated versions.^49^ This research represents a systematic attempt to improve the availability of outcome measures in Cambodia by examining the psychometric properties of the Khmer version of the LCI-5.^19^

The LCI-5 is a commonly reported outcome measure in studies involving major lower-limb amputations^10,51^ and has been translated to multiple languages.^12,14,16-18^ It has been incorporated into the British Association of Chartered Physiotherapists in Limb Absence Rehabilitation toolbox of outcome measures^52^ and as a quality indicator in the Swedish national register for amputation and prostheses (SwedAmp).^53^ The LCI-5 was considered relevant for the Cambodian context as it was considered easy to administer, gives the client's perspective regarding their mobility, and includes items that have previously identified as challenging for Cambodian prosthesis and orthosis users.^6^ Although the LCI-5 was originally developed to be used with prosthesis users, previous work has adapted the instrument so that it could be used with lower-limb orthosis users.^19^ This group indicated that psychometric testing was required before the instrument was put to use with this population of clients. For this reason, this study performed one set of analyses with the original target population (prosthesis users) and a second analysis to determine if the instrument may be relevant to use with lower-limb orthosis users.

The original LCI, developed with a 4-point response scale, was identified as having issues with skewed scores due to ceiling effects.^54,55^ The LCI-5 subsequently developed to include 5 response alternatives. This 5-point scale greatly reduced the percentage of top scores and allowed for better distinction among grades of mobility.^12^ Our descriptive statistics for the Khmer sample suggest that the Khmer version of the LCI-5 is prone to ceiling effects with high mean scores, low variance, negative skewness, and high kurtosis. This was particularly evident in items 1 to 5, a result that is consistent with findings reported when the Persian version of the LCI-5 was tested with a sample of Iranian prosthesis users.^18^ The observed ceiling effects are likely due differences in the physical status of clients who can access and receive prosthetic or orthotic devices and are included in the Cambodian and Iranian samples. In comparison with other countries in which the LCI-5 have been psychometrically tested, for example, Italy,^12^ Canada,^56^ Sweden,^16^ and Finland,^17^ where prosthetic and orthotic services are highly accessible, access to services in Cambodia and Iran is seriously limited.^6,57^ It is therefore likely that people with more serious mobility limitations in their countries are unable to access services and are not represented in the samples included in psychometric testing. As such, it is important to recognize the limitations of the Khmer version of the LCI-5 and recognize that it is not sensitive enough to distinguish among respondents at the upper end of the scale. Addressing these ceiling effects will require revisions to the current Khmer version of the instrument, including the addition of more challenging mobility items that can better differentiate between individuals with higher levels of mobility.

The factor structure of the LCI-5 has been addressed in the English and Finnish versions of the outcome measure.^17,56^ The original English version identified 2 explanatory factors representing basic (7 items) and advanced (7 items) activities,^56^ while the Finnish version loaded on only one factor,^17^ suggesting that this version may not be sensitive enough to distinguish between different mobility levels. In addressing our aim to measure structural validity in the Khmer version of LCI-5, we identified a two-factor model that was considered to have adequate fit for both prosthesis and orthosis users. It is important to recognize, however, that items within our 2-factor model differed from those proposed in the original English version.^56^ Our results saw items involving bending to get up from the floor or to pick up objects, considered as advanced activities in the original version, loading on the same factor as the basic activities of walking in the house and walking on even ground. Differences across cultures are likely to explain the contrasting factor structures between the English and Khmer versions. For example, within Cambodian culture, sitting on the floor is rooted in daily life and religious practices, while in western contexts, people are not accustomed to sitting on the floor and may even find it uncomfortable. It is subsequently likely that activities, such as bending and getting up from the floor, are practiced much more frequently by the Cambodians and are thus considered easier to perform. All items loading on factor 2 in the Khmer version were found within the advanced subcategory of the original English version.^56^

The original version of the LCI-5 distinguished between going up and down stairs by separating the activities into different items (e.g., item 8, go up the stairs with a handrail, and item 9, go down the stairs with a handrail).^56^ Our analysis indicated a high degree of covariance between these items, suggesting that they are conceptualized in such a way that they overlap or that they are measuring the same underlying ability. To address this covariance, we removed all items referring of walking upstairs and retained items involving walking downstairs. In future modifications to the Khmer version of the LCI-5 or indeed in development of other mobility measures that will be used in the Cambodian context, we suggest grouping the activities into one item “stair walking.”

Few outcome measures are available to assess the mobility status of lower-limb orthosis users.^58^ In an attempt to address this issue, the Khmer version of the LCI-5 was translated and adapted to make this possible.^19^ Our results showed that the modified 2-factor structure was a good fit when tested with a sample of orthosis users and, taking into consideration potential ceiling effects, may be useful as a measure of mobility with users of lower-limb orthoses.

The results from this study suggest that, in the clinical setting, the current version of the Khmer LCI-5 would be most appropriate to apply when assessing prosthetic and orthotic interventions on new users or people with suspected mobility limitations. Owing to ceiling effects, it is not an appropriate tool to apply to established users with high levels of mobility. In the absence of a suitable PROM for measuring mobility in Cambodian clients with high levels of function, clinicians may be tempted to turn to performance-based measures, which can be based on an objective measurements (e.g., 6-minute walk test^59^; timed up and go test^60^) or scored qualitative assessments (e.g., Berg Balance Scale^61^). Although performance measure offers a very good alternative, it is important to recognize that they too can be subject to ceiling effects^62^ and would also need to undergo validation with Cambodian prosthetic and orthotic users before being implemented into routine clinical care.

## Limitations and future research

When performing the CFA in this study, post hoc modifications were required to be made to achieve an acceptable model fit.^32^ This may have resulted in a model that is overly fitted to our samples and can reduce the generalizability to other prosthesis and orthosis users. One way to address this issue in future studies could be to use a Bayesian Lasso method in which the entire residual covariance matrix for all observed measures is modelled as a sparse structure.^63^

This research focused heavily on evaluating the structural validity of the LCI-5, and it is acknowledged that some psychometric characteristics still need to be assessed. Test-retest reliability of the measure is one issue that remains to be evaluated. The extent to which the Khmer version of the LCI-5 can be used to predict client outcomes is also of interest for future investigations. Reliability and predictive validity testing would both require the collection of longitudinal data.

Within the context of this research, it is important to recognize that the factor structure for basic and advanced activities in the Khmer version of the LCI-5 differ from those in the original English version. This suggests that constructs in the English version may manifest differently in the Khmer version and limits the extent to which the instrument can be used to make cross-cultural comparisons. Future research should focus on developing instruments that function equivalently across different cultures. Here, it will be important to consider the unique cultural, linguistic, and social factors associated with different regions of the world. We suggest that addressing the issue will require a participatory design which includes members from different cultural contexts who can ensure that items resonate with the lived experiences of respondents from their respective countries.

As the aim of this study was to determine psychometric properties of the culturally validated Khmer version of the LCI-5, no potentially new items were added or tested. Future research should, however, explore modifications to the Khmer version of the LCI-5, which can address the observed issues with ceiling effects. This could be done by adding or refining existing items that could help differentiate among high-performing individuals in the Cambodian context. Such items could include carrying heavy loads, riding and dismounting a moto or tuk-tuk, crossing busy streets or navigating crowded markets, or kneeling to pray.

We recommend that future work makes use of item response theory^64^ to evaluate the performance of specific items across the mobility spectrum, assisting to identify items that do not adequately discriminate at the higher level of the scale.

## Conclusion

It is commonly accepted that outcome measures are essential for good clinical practice; however, the use of such measures is seriously limited by their lack of availability in local languages. This is particularly evident in many low- and middle-income countries.^49^ This study represents a small step toward improving the availability of outcome measures for prosthetic and orthotic research and practice in Cambodia by validating the Khmer version of the LCI-5. The modified 2-factor model identified in this study will support research initiatives aimed at identifying variables having an impact on the mobility outcomes of prosthetic and orthotic users as well as evaluating outcomes for individual clients with mobility limitations. Additional work is required to address ceiling effects associated with the Khmer version of the LCI-5.

## Funding

The author(s) disclosed receipt of the following financial support for the research, authorship, and/or publication of this article: This research was funded by The Swedish Research Council (grant # 2001-04299).

## Declaration of conflicting interest

The author(s) disclosed no potential conflicts of interest with respect to the research, authorship, and/or publication of this article.

## Supplemental material

Supplemental material for this article is available in this article. Direct URL citation appears in the text and is provided in the HTML and PDF versions of this article on the journal's Web site (www.POIjournal.org).

## Supplementary Material
