# Supplementary material for: Psychometric properties of the Khmer version of the locomotor capabilities index (LCI-5) for lower-limb prosthesis and orthosis users
Source: Prosthet Orthot Int. 2025 Oct 23;50(5):550–9. doi: 10.1097/PXR.0000000000000504 (PMC13645411; doi:10.1097/PXR.0000000000000504)
Supplement: Supplementary file 1 [file poi-50-550-s001.docx]

**Supplementary data**

Supplementary data 1 - results of parallel analysis (n=173)

| Root | Real data | Mean | Percentile |
| --- | --- | --- | --- |
| 1 | 6.281 | 1.510 | 1.627 |
| 2 | 2.217 | 1.385 | 1.471 |
| 3 | 1.133 | 1.289 | 1.355 |
| 4 | 0.931 | 1.209 | 1.270 |
| 5 | 0.806 | 1.138 | 1.194 |
| 6 | 0.627 | 1.071 | 1.119 |
| 7 | 0.600 | 1.010 | 1.055 |
| 8 | 0.451 | 0.950 | 0.997 |
| 9 | 0.333 | 0.889 | 0.937 |
| 10 | 0.209 | 0.833 | 0.879 |
| 11 | 0.157 | 0.773 | 0.820 |
| 12 | 0.119 | 0.715 | 0.767 |
| 13 | 0.073 | 0.651 | 0.703 |
| 14 | 0.062 | 0.576 | 0.637 |

Supplementary data 2 - Minimum average partial test (MAP) n=173

|  | Eigenvalues | Proportion of Variance | Cumulative prop-variance |  |
| --- | --- | --- | --- | --- |
| Factor 1 | 6.28 | 0.45 | 0.45 |  |
| Factor 2 | 2.22 | 0.16 | 0.61 |  |
| Factor 3 | 1.13 | 0.08 | 0.69 |  |
| Factor 4 | 0.93 | 0.07 | 0.75 |  |
| Factor 5 | 0.81 | 0.06 | 0.81 |  |
| Factor 6 | 0.63 | 0.04 | 0.86 |  |
| Factor 7 | 0.60 | 0.04 | 0.90 |  |
| Factor 8 | 0.45 | 0.03 | 0.93 |  |
| Factor 9 | 0.33 | 0.02 | 0.96 |  |
| Factor 10 | 0.21 | 0.01 | 0.97 |  |
| Factor 11 | 0.16 | 0.01 | 0.98 |  |
| Factor 12 | 0.12 | 0.01 | 0.99 |  |
| Factor 13 | 0.07 | 0.01 | 1.00 |  |
| Factor 14 | 0.06 | 0.00 | 1.00 |  |

Supplementary data 3 – Scree plot

Supplementary data 4 – Khmer version of LCI-5 for prosthesis users

Supplementary data 5 – Khmer version of LCI-5 for orthosis users
